# Assessing Nutritional Parameters of Brown Bear Diets among Ecosystems Gives Insight into Differences among Populations

**DOI:** 10.1371/journal.pone.0128088

**Published:** 2015-06-17

**Authors:** Claudia López-Alfaro, Sean C. P. Coogan, Charles T. Robbins, Jennifer K. Fortin, Scott E. Nielsen

**Affiliations:** 1 Department of Renewable Resources, University of Alberta, 751 GSB, Edmonton, T6G 2H1, AB, Canada; 2 Departamento de Ciencias Ambientales y Recursos Naturales Renovables, Universidad de Chile, Av. Santa Rosa, 11315, Casilla 9206, Santiago Chile; 3 School of Biological Sciences and the Charles Perkins Centre, University of Sydney, Sydney, NSW 2006, Australia; 4 School of the Environment and School of Biological Sciences, Washington State University, Pullman, WA, United States of America; 5 School of Biological Sciences, Washington State University, Pullman, WA, United States of America; Michigan Technological University, UNITED STATES

## Abstract

Food habit studies are among the first steps used to understand wildlife-habitat relationships. However, these studies are in themselves insufficient to understand differences in population productivity and life histories, because they do not provide a direct measure of the energetic value or nutritional composition of the complete diet. Here, we developed a dynamic model integrating food habits and nutritional information to assess nutritional parameters of brown bear (*Ursus arctos*) diets among three interior ecosystems of North America. Specifically, we estimate the average amount of digestible energy and protein (per kilogram fresh diet) content in the diet and across the active season by bears living in western Alberta, the Flathead River (FR) drainage of southeast British Columbia, and the Greater Yellowstone Ecosystem (GYE). As well, we estimate the proportion of energy and protein in the diet contributed by different food items, thereby highlighting important food resources in each ecosystem. Bear diets in Alberta had the lowest levels of digestible protein and energy through all seasons, which might help explain the low reproductive rates of this population. The FR diet had protein levels similar to the recent male diet in the GYE during spring, but energy levels were lower during late summer and fall. Historic and recent diets in GYE had the most energy and protein, which is consistent with their larger body sizes and higher population productivity. However, a recent decrease in consumption of trout (*Oncorhynchus clarki*), whitebark pine nuts (*Pinus albicaulis*), and ungulates, particularly elk (*Cervus elaphus*), in GYE bears has decreased the energy and protein content of their diet. The patterns observed suggest that bear body size and population densities are influenced by seasonal availability of protein an energy, likely due in part to nutritional influences on mass gain and reproductive success.

## Introduction

Among wide-ranging species, conspecific animals occupying different habitats often differ in body size, reproductive traits (e.g. age of first litter, litter interval, and litter size) and density among populations [1–3]. These differences in life history traits among populations may be genetic or phenotypic, and are frequently associated with differences in food availability, either quantity or quality [1, 4, 5]. Food habits and nutritional studies are among the first steps used to understand wildlife-habitat relationships. Generally, these studies use fecal analysis to describe diet composition of a species within a population. While such studies sometimes include nutritional information of foods (e.g., energy and protein content), they often lack a nutritional evaluation of the complete diet. Such information is necessary to determine key nutritional elements and assess how they influence life history traits including foraging behavior, reproductive success, and population dynamics. An explicit evaluation of the nutritional parameters of an animal’s diet is essential to comprehend nutritional mechanisms affecting individual fitness [6, 7] and habitat selection or foraging patterns [8] in different ecosystems.

Brown bears (*Ursus arctos*) are widely distributed and found across a variety of habitats [7, 9]. Nutritional differences in the habitats occupied by brown bears often lead to variations in body and litter size, inter-litter interval, and population densities [3, 4, 10, 11]. One reason for such diet variation is that brown bears are flexible omnivores [12], and their diets can range from largely carnivorous to largely herbivorous [4, 7, 13]. Given the demands of lengthy annual hibernation, the diet of brown bears during the active season is critical to their survival and reproductive success, which depends on both maternal fat [14, 15] and lean mass reserves [16] before denning. For brown bears, lean mass growth occurs primarily during spring and early summer, while fat accumulation occurs mostly during summer and early fall before hibernation [4, 17, 18].

In general, interior brown bear populations in North America are composed of smaller, more herbivorous bears than coastal populations with access to salmon (*Oncorhynchus spp*.; [4, 10]). Population densities and reproductive success are also lower in inland populations compared to those found on the coast [1, 3, 11, 18]. Among interior populations, such as along the Rocky Mountains, noticeable differences can be seen between populations. Alberta’s bear populations occur along the eastern slopes of the Canadian Rocky Mountains and adjacent Foothills to the east [19]. Alberta brown bear sub-populations differ in densities (5–18 bears/1000km^2^) and body condition [20–22] with spring body mass for females of 109–129 kg [1, 23]. Brown bears have been designated as a provincially threatened species in Alberta, in part due to their low reproductive rate and slow recovery [20].

In contrast, the Flathead River (FR) ecosystem (west slopes of the Canadian Rockies) is located in the southeast part of British Columbia adjacent to south-western Alberta and sustains a productive brown bear population. Bear densities there are among the highest recorded for interior populations, ranging from 25 to 55 bears/1000km^2^, but body masses of females range between 97–114 kg [18, 24].

Further south along the Rocky Mountains, the Greater Yellowstone Ecosystem (GYE) supports a productive population with spring and summer female body masses of 112 kg (SE = 5; [25]) and densities of 13–16 bears/1000km^2^ [26, 27]. The GYE population has increased from 135 individuals in 1983 [28] to 593 individuals in 2010 [29]. Despite this recovery, the GYE bear population faces some recent nutritional changes. Cutthroat trout (*Oncorhynchus clarki*) populations in Yellowstone Lake, which once made up an important part of the diets of some bears [30], have markedly declined due to the introduction of non-native lake trout (*Salvelinus namaycush*) and “whirling disease” (*Myxoblus cerebralis*; [31–34]). Whitebark pine (*Pinus albicaulis*) nuts, a key food that affects reproductive success [35, 36], has also declined due to whitebark pine blister rust (*Cronoartium ribicola*) and mountain pine beetles (*Dendroctonus ponderosae*; [33, 37, 38]).

Changes in management of the GYE ecosystem have previously affected food availability for brown bears, such as the increase in elk population due to wolf extirpation in the early 20^th^ century [39] and the garbage dump closures in the late 1960s and early 1970s [12, 40]. Despite this, the proportion of meat in the diets of female grizzly bears living in parts of the GYE appears to be stable since the late 1970s [17, 33, 41]. However, male GYE grizzly bears show a decrease in meat consumption between 1997–2000 to 2007–2009 [17] with both those periods showing lower meat consumption than eco-system wide estimates from 1977–1996 [41]. Due to the reduction in the elk population that began in approximately 1995 coincident with wolf re-establishment [39, 42–44], we expected the meat content of female diets should decrease and, thereby, potentially reduce reproductive success and population productivity.

The seasonal diets of bears in Alberta, FR, and GYE have been determined via fecal analysis, and, with the exception of the older studies, have incorporated brown bear-specific correction factors (CFs) to account for differences between the volume of food residues found in the scats and the volume of food ingested [45, 46]. Despite CFs for a wide range of food items, most studies often use a single CF for groups of foods, where the CF is often chosen conservatively (i.e., an underestimate). There is, however, a large variation in the CF used for terrestrial meat compared to other foods (e.g. 10-fold higher) that depends on the proportion of skin, hair, and bones consumed and the capacity to distinguish among these components during the fecal analysis [46]. Choosing a single CF for terrestrial meat, therefore, may not accurately estimate its dietary prevalence. However, applying a range of CFs to meat residues found in bear scats may be intractable in most cases.

The digestive and metabolic capacity of brown bears in relation to certain foods also has been investigated [47], allowing for more complex examinations of dietary relationships of brown bears. Previous studies have integrated this physiological information with food habits studies in different ecosystems, helping us understand how food resources influence life history traits in bears by illustrating: 1) the influence of dietary meat intake on body size and population density [1, 4, 18, 48]; 2) the importance of primary productivity and seasonality on bear reproductive traits, such as age of primiparity and inter-birth interval [1], and population densities [10, 11]; and 3) the significance of digestible energy and other nutrients on the patterns of food habits of brown bears [7]. Quantitative methods, however, to evaluate and compare between nutritional parameters of ecosystem-specific bear diets have not been explored.

In this study, we developed a dynamic model integrating food habits and nutritional information to assess nutritional parameters of brown bear diets among three interior North American ecosystems, thereby allowing for nutritional comparisons between ecosystems. Our model addresses five objectives that include: 1) quantifying the differences in the amount of digestible protein and energy of bear diets in west-central Alberta, the FR, and both the historical (1977–1987) and recent (2007–2009) GYE; 2) relating the nutritional patterns to differences in individual body size and population densities; 3) determining the proportion of total dietary energy and protein provided by different foods within each ecosystem; 4) assessing the impact of dietary shifts in the GYE on energy and protein consumption; and 5) evaluating the impact of using different CFs on the nutritional evaluation of bear diets.

We hypothesized that because fat and lean mass accumulation are positively related to reproductive success, the amount of digestible protein in spring and early summer diets and digestible energy of late summer and fall diets should be higher in the FR and GYE than in west-central Alberta. Based on differences in individual body size, we expect protein to be lower in the FR than in the GYE. Due to the recent decrease in trout [30, 33] and pine nuts in the diets of GYE bears [37, 38], differences in the amount of protein and energy between historical and recent diets should be apparent. Because male bears in the GYE have recently shown reductions in meat consumption [17, 33, 41], we expected differences in male diets to be the most noticeable.

## Methods

### Brown bear food habits

Four published brown bear food habits studies were used to quantify ecosystem-specific nutritional quality [30, 33, 49, 50]. Studies were selected because they represent a natural gradient (North–South) in interior ecosystems inhabited by brown bears along the North American Rocky Mountains, and food habits have been described on a volumetric basis which allows for the application of CFs to account for differences in quantification of food residues (“volumetric weight” of foods in the scat are used to determine diet composition, in contrast to “frequency of occurrence” estimates which are used to study food selection). Also, these studies span the active period from spring to fall, which allow us to examine temporal patterns in the nutritional quality of bear diets. Bear food habits in these studies represent an average diet across years in which scat was collected. We therefore do not consider inter-annual variations in diets.

### West-central Alberta food habits

In west-central Alberta, noticeable differences in diet were exhibited between bears living in the Mountain versus Foothills and were therefore separated as in Munro, Nielsen [50]. Bear food habits presented in Munro, Nielsen [50] were based on 665 scats of 18 brown bears collected between April and October 2001–2003. The diets of Foothills bears in Munro, Nielsen [50] were examined from late April to early October in bi-monthly periods, while the diets of Mountain bears were examined from late April to late September. Therefore, we extended the diet estimates for the Mountain bears to early October based on the authors’ suggestion that bear diets during that time were largely composed of roots and green vegetation.

### Flathead river food habits

Information on food habits for the FR ecosystem of southeastern British Columbia, Canada was obtained from McLellan and Hovey [49]. This study was based on 1100 scats collected between April and November 1978–1991 from 77 radio-collared brown bears. Diet descriptions extended from early April to early November, which we divided into bi-monthly periods, but we only use the period between late April and early October to compare with the other studies. The McLellan and Hovey [49] study was conducted before correction factors were developed to relate fecal proportions to actual dietary proportions [46]. Thus, we corrected fecal proportions to dietary dry matter proportions using the corresponding CFs from Hewitt and Robbins [46] as applied in Fortin, Schwartz [33] (Table 1). Because the study of McLellan and Hovey [49] was conducted more than two decades ago, it might be considered an historical condition of the bear food habits in the FR ecosystems.

**Table 1:** Correction factors (CFs) used in the model.

Food item	Fixed CFs	CFs range
**Graminoids**	0.24	0.23–0.25
**Horsetail**	0.16	0.14–0.19
**Sedges**	0.18	0.16–0.20
**Dandelion**	0.32	0.29–0.36
**Clover**	0.33	0.29–0.38
**Elk thistle**	0.24	0.19–0.29
**Green vegetation** ^**1**^	0.26	0.14–0.43
**Forbs** ^**2**^	0.26	0.14–0.43
**Large mammals** ^**3**^	3.0	1.37–12.5
**Small mammals** ^**4**^	4.0	3.80–12.5
**Roots** ^**5**^	1.0	0.32–1.53
**Hedysarum spp.**	0.35	0.32–0.38
**Fruits**	1.2	0.50–2.24
**Insects**	1.1	0.88–1.44
**Pine nuts**	1.54	1.23–1.85
**Trout**	40.8	39.5–42.3
**False-truffles**	1.1^6^	0.88–1.44^6^

^1^ Green vegetation includes all species not defined in the previous categories.

^2^ Includes species defined as forbs

^3^ Large mammals include elk, bison, white-tailed deer, and moose.

^4^ Small mammals include rododent, squirrels.

^5^ Roots include *hedysarum* spp., biscuit roots, and select cultivated root vegetables (carrot, yam, and sweet potato).

^6^ We applied the same range value on Insects.

We ran the model twice, one using a fixed CFs for each food groups, and a second allowing the CFs to vary randomly between the ranges presented in this table.

### Greater Yellowstone ecosystem food habits

Two diet studies were used to characterize the historical (1977–1987) and recent (2007–2009) diets of brown bears in GYE. The first study by Mattson, Blanchard [30] included Yellowstone National Park and surrounding National Forest and was based on 3,423 scats from 96 radio-collared bears. Diet descriptions extended from April to October by month, which we divided into bi-monthly periods for modelling. As in the Flathead study, fecal proportions were corrected to dietary dry matter proportions using the above CFs (Table 1; [46]).

The most recent GYE food habit study [33] included only the area immediately surrounding Yellowstone Lake. Thus, the comparison between the two studies [30, 33] may include changes over both time (e.g., 1977–1987 and 2007–2009) and space (e.g., the larger Yellowstone ecosystem as compared to the area surrounding Yellowstone Lake). Diet estimates for GYE bears were divided into male and female, each containing both adults and subadults. Scats were collected between 2007 and 2009 (n = 778). Food habits descriptions extend from May to September for males and to October for females in monthly periods ([33]; Fortin unpublished), hence we extended the diet estimates from late April to early October based on the field observations of researchers. For male bears we assumed that late-April diets were ungulates and graminoids. For early October, we assumed that bear diets were ungulates, graminoids, and a small fraction of false-truffles (mushrooms).

### Food categories

Bear foods identified in the Alberta, FR, and GYE studies were grouped into eight dietary (food) categories: green vegetation, berries, roots, ants, terrestrial meats, pine nuts, trout, and false-truffles (Table 2).

**Table 2:** Nutritional information used to obtain the digestible energy and protein in one kilogram of fresh diet. Values were estimated using data presented in Table 3.

	DM (%)	DMDig (%)	GrossE (kcal/g)	EDig (%)	PC (%)	PDig (%)	References
**Vegetation spring**	20.1 (5.1) [3]	36.6 (8.8) [2]	4.5^1^ (0.3) [/]	41.3 (8.2) [3]	25.9 (3.7) [8]	74.5 (0.6) [8]	[47, 51–53], Coogan unpublished
**Vegetation summer**	21.5 (8.9) [9]	27.8 (12.2) [15]	4.5 (0.3) [9]	35.3 (12.4) [12]	19.4 (6.0) [23]	66.0 (13.0) [23]	[53], Coogan unpublish, Fortin unpublished
**Vegetation fall**	28.3 (9.6) [5]	18.4 (7.7) [8]	4.5 (0.1) [3]	24.3 (11.7) [5]	14.7 (5.8) [11]	61.2 (9.0) [11]	[51, 53], Coogan unpublished, Fortin unpublished
**Berries**	15.1 (2.8) [12]	63.9 (10.9) [8]	4.3 (0.2) [7]	60.0 (10.1) [6]	4.6 (0.8) [7]	14.8 (4.1) [4]	[47, 54, 55, 56], Fortin unpublished
**Roots (Alberta and Flathead)**	22.0 (9.0) [8]	44.3 (10.1) [3]	4.0 (0.2) [2]	39.2 (3.2) [2]	Coogan, et al. (2012)^3^	60.6 (3.4) [4]	[47, 55–57, 58], Fortin unpublished
**Roots (GYE)**	22.0 (9.0) [8]	44.3 (10.1) [3]	3.9 (0.2) [10]	58.1 (12.3) [9]	8.9 (3.1) [16]	44.9 (15.2) [15]	[47, 53, 55, 56, 57], Fortin unpublished
**Ants**	27.4 (2.8) [3]	76.6 (9.8) [3]	4.7 (1.6) [12]	18.7 (1.3) [3]	46.3 (14.2) [12]	77.6 (7.2) [12]	[55, 59, 60], Coogan unpublished
**Terrestrial meat**	27.0 (3.5) [11]	87.5 (8.0) [3]	5.2 (1.0) [11]	92.5 (3.4) [9]	72.9 (15.5) [17]	88.2 (3.5) [17]	[47, 53, 56], Fortin unpublished
**Pine nuts**	93.2 (4.3) [4]	42.9 (18.6) [3]	6.5 (0.4) [6]	49.7 (0.4) [2]	12.4 (1.9) [6]	36.1 (18.3) [6]	[47, 53, 55, 56, 61], Fortin unpublished
**Trout**	27.2 (1.7) [4]	89.8 (8.9)^2^ [/]	5.4 (0.7) [4]	94.5 (9.5)^2^ [/]	71.0 (4.0) [4]	91.5 (3.7) [4]	[47, 56], Fortin unpublished
**False-truffles**	59.3 (5.9) ^2^ [/]	81.5 (8.2) [2]	4.7 (0.5)^2^ [2]	51.1 (5.1)^2^ [/]	18.3 (6.2) [3]	69.2 (6.9)^2^ [/]	[47, 53, 61], Fortin unpublished

DM (%) = Dry matter (% of fresh matter); DMDig (%) = Digestible dry matter; GrossE (kcal/g) = Gross energy; EDig (%) = Energy digestibility; PC (%) = Protein content; PDig (%) = Protein digestibility. DMDig, GrossE, EDig, PC and PDig in a dry matter basis.

In parenthesis is standard deviation. In brackets is sample size.

^1^: GrossE for vegetation spring was assumed to be the same than vegetation in summer.

^2^: Standard deviation estimated as the 10% of average value.

^3^: Nutritional information for roots in Alberta ecosystems and Flathead was extracted from figure two in Coogan, Nielsen [58].

The green vegetation category included 13 species of grasses, forbs and horsetails (Table 3) and values obtained from USDA National Nutrient Database for spinach (*Spinacia oleracea*) and lettuce (*Lactuca sativa*). Nutritional values for green vegetation were estimated for three phenological stages: Spring (from 15 April to 31 May); Summer (from 1 June to 31 July); and Fall (1 August to 15 October). To match the plant phenology in the higher elevation Mountain ecosystems in Alberta, the Spring stage was extended until June 15.

**Table 3:** Nutritional information for different bear food items.

	Foods items	DM (%)	DMDig (%)	GrossE (kcal/kg)	EDig (%)	PC (%)	PDig (g)	PDig (%)	TDF (%)	Reference
**VEGETATION ANNUAL**	Alfalfa leaves & stems (*Medicago sativa*)		18.5 (1) [2]	4158	23.4 (0.8) [2]	22.1 (2.8) [2]	16.0^1^ (2.5) [2]	72.4	59.6 (0.7) [2]	Coogan [62]
	Clover leaves & stems (*Trifolium* spp)		20.0 (3.6) [10]	4382 (270) [14]	24.7 (3) [10]	19.4 (1.3) [21]	13.6^1^ (1.8) [20]	70.1	58.5 (2.6) [10]	Coogan [62]
	Clover flower (*Trifolium* spp)		38.9		40.4 [1]	27.1 [1]	20.4^1^ (0.4) [1]	75.3	44.9 [1]	Coogan [62]
	Clover white	14.1	46.1 (0.4)	5311	51.1 (0.4)	30.2	23.2	76.9 (0.1)	42.0	Pritchard and Robbins [47]
	Cow parsnip leaves & stems (*Heracleum lanatum*)		11.3 (3.8) [8]	3640 (570) [7]	17.4 (3.1)	13.9 (2.2) [8]	8.7 (1.9) [8]	62.6	64.8 (2.7) [8]	Coogan [62]
	Dandelion (foliage and flower)				45.6 (5.8) [46]	19.2 (6.7) [2]	13.5^1^	70.1	14 (5.2) [2]	Mattson, Barber [53]
	Dandelion flower (*Taraxacum officinale*)		46.8 (1.4) [3]		46.9 (1.2) [3]	17.0 (0.3) [6]	11.5^1^ (0.4) [6]	67.6	39.2 (1) [3]	Coogan [62]
	Dandelion leaves & stems (*Taraxacum officinale*)		48.0 (2.9) [6]	3828 (70) [3]	47.9 (2.4) [6]	17.0 (2) [11]	11.5^1^ (2.1) [11]	67.6	38.4 (2.1) [6]	Coogan [62]
	Elk thistle (stem)				18.8 (11.2) [16]	4.1 (1.6) [16]	0.2	3.7	27.8 (4.5) [16]	Mattson, Barber [53]
	Horsetail				32.0 (8.6) [27]	13.3 (5.6) [26]	8.3	62.1	20.8 (4.8) [27]	Mattson, Barber [53]
	Horsetails (*Equisetum arvense*)		31.8 (3.3) [2]		34.4 (2.7) [2]	20.4 (2.2) [8]	14.5 (1.9) [8]	71.1	50 (2.4) [2]	Coogan [62]
	Horsetails (*Equisetum sylvaticum*)		46.3 (5.4) [1]		46.3 (4.5) [1]	20.0 (4.6) [2]	14.2 (4.4) [2]	70.8	39.7 (3.9) [1]	Coogan [62]
	Spring beauty (foliage and flower)				48.7 (7.4) [12]	25.4 (5.8) [11]	13.7 (3.3) [12]	53.9	13.7 (3.3) [12]	Mattson, Barber [53]
	Dandelion greens, raw (*Taraxacum officinale*)	14.4		3125		18.8	9.2^1^	48.9	3.5	USDA [56]
	Fireweed, leaves, raw (*Epilobium angustifolium*)	29.2 [2]		3525		16.1 [2]	6.5^1^	40.4	10.6 [2]	USDA [56]
	Spinach, raw (*Spinacia oleracea*)	8.6 [1]		2674		33.3 [9]	23.8^1^	71.6	2.2 [1]	USDA [56]
	Lettuce green leaves, raw (*Lactuca sativa* var. crispa)	5.0 [14]		2988		27.1 [8]	17.6^1^	64.0	1.3 [4]	USDA [56]
**VEGETATION SPRING**	Clover (May)					30.5 [1]	23.4 [1]	76.7		Coogan, Raubenheimer [52], Coogan (unpublished)
	Clover (Spring)				52.8 (3.5) [4]	25.7 (2) [4]	19.2	74.6	12.6 (1.3) [4]	Mattson, Barber [53]
	White clover (spring-early summer; *Trifolium ripens giganteum*)	15 (1.4) [12]	45.4 (2.9) [12]			27.6 (1.5) [12]	20.9	75.6	40.1 (4.2) [12]	Rode, Robbins [51]
	Dandelion (May)					29.8 [1]	22.8 [1]	76.5		Coogan, Raubenheimer [52], Coogan (unpublished)
	Graminoids (Spring, emergence—May15)				33.9 (8.1) [24]	20.7 (4.8) [24]	14.8	71.4	21.2 (3.9) [24]	Mattson, Barber [53]
	Grasses (spring-early summer; *Poa pratensis*, *Phleumpratense*, *Bromus gracilis*)	25.2 (1.2) [12]	27.8 (4.6) [12]			20.5 (2.4) [12]	14.6	71.2	52.4 (4.1) [12]	Rode, Robbins [51]
	Horsetails (May)				37.2	26.8 [1]	20.2 [1]	75.4	47.6 [1]	Coogan, Raubenheimer [52], Coogan (unpublished)
**VEGETATION SUMMER**	Clover (early hyperphagia)				38.6 (8.4)	20.3 (3.3)	14.4	71.0	19.2 (4.2)	Mattson, Barber [53]
	Clover (estrus)				42.7 (12)	21.5 (2.7)	15.5	72.0	16.7 (6.1)	Mattson, Barber [53]
	Clover (July)		23.3 (14.1)		27.4 (11.7) [5]	19.5 (3.5) [7]	13.7 (3.1) [7]	70.3	56.1 (10.1) [5]	Coogan, Raubenheimer [52], Coogan (unpublished)
	Clover (June)					32.7 (5.6) [2]	25.4 (4.9) [2]	77.7		Coogan, Raubenheimer [52], Coogan (unpublished)
	Cow parsnip (July)		7.5 (3.6)		14.3 (3) [3]	15.1 (1.9) [3]	9.8 (2.1) [3]	64.9	67.5 (2.6) [3]	Coogan, Raubenheimer [52], Coogan (unpublished)
	Cow parsnip (June)		21.9		26.2 [1]	26.8 [1]	20.2 [1]	75.4	57.1 [1]	Coogan, Raubenheimer [52], Coogan (unpublished)
	Dandelion (June)		52.8 (2.7)		51.9 (2.2) [4]	19.9 (2.2) [4]	4.1 (2) [4]	70.8	34.9 (1.9) [4]	Coogan, Raubenheimer [52], Coogan (unpublished)
	Dandelion (July)		40.7 (3.8)		41.8 (3.2) [2]	16.1 (5.4) [2]	10.7 (4.7) [2]	66.5	43.6 (2.7) [2]	Coogan, Raubenheimer [52], Coogan (unpublished)
	Fireweed (early hyperphagia)				56.9 (4.6)	23 (4.5)	16.8	73.1	9.2 (1)	Mattson, Barber [53]
	Fireweed (estrus)				43.6	15.8	10.5	66.2	8.4	Mattson, Barber [53]
	Graminoids (estrus, May15—July 15)				31.4 (9.1) [37]	20.1 (5.4) [36]	14.2	70.9	23.0 (4.6) [37]	Mattson, Barber [53]
	Graminoids (early hyperphagia, July16 –August 30)				17.5 (12.4) [5]	5 (3.1) [5]	0.95	18.9	29.4 (5.9) [5]	Mattson, Barber [53]
	Horsetails (July)		28.5 [1]		31.7 [1]	21.9 (2.2) [2]	15.8 (2) [2]	72.1	52.4 [1]	Coogan, Raubenheimer [52], Coogan (unpublished)
	Horsetails (June)					29.6 [1]	22.7 [1]	76.7		Coogan, Raubenheimer [52], Coogan (unpublished)
	Angelica	17.7 (0.9) [2]	26.24^2^	4878 (325) [2]		15.0 (6.1) [2]	9.8^1^	65.0	54.0 (11.8) [2]	Fortin (unpublished)
	Elk thistle	8.6 (1.2) [4]	36.4^2^	3804 (23.4) [4]		7,1 (2.6) [4]	2.8^1^	39.4	46.7 (2.7) [4]	Fortin (unpublished)
	Elymus	42 (2.7) [3]	6.0^2^	4434 (147) [3]		8.8 (5.1) [3]	4.3^1^	48.8	68.5 (0.4) [3]	Fortin (unpublished)
	Fireweed	20.1 (4.3) [4]	26.8^2^	4482 (94.5) [4]		19.2 (7.4) [4]	13.5^1^	70.1	53.6 (2.9) [4]	Fortin (unpublished)
	Cow parsnip	17.2 (6.3) [3]	33.7^2^	4471 (549) [3]		19.2 (13.8) [3]	13.5^1^	70.1	48.6 (4.4) [3]	Fortin (unpublished)
	Fern-leaf lovage	17.8 (2.9) [3]	28.6^2^	4457 (241) [3]		19.7 (3.9) [3]	13.9^1^	70.5	52.3 (0.5) [3]	Fortin (unpublished)
	Timothy	28.4 (3.5) [2]	12.8^2^	4828 (82) [2]		17.4 (12.1) [2]	11.9^1^	68.2	63.7 (9.4) [2]	Fortin (unpublished)
	Dandelion	16.8 (1.6) [7]	40.7^2^	4442 (609) [7]		15.1 (3.1) [7]	9.8^1^	65.2	43.6 (5.5) [7]	Fortin (unpublished)
	Clover	25.2 (4.6) [3]	30.7^2^	4746 (35) [3]		25.3 (4.6) [3]	18.8^1^	74.4	50.8 (1.5) [2]	Fortin (unpublished)
**VEGETATION FALL**	Clover (August)		16.6 (9.4)		21.9 (7.8) [4]	16.1 (1.5) [8]	10.7 (1.4) [8]	66.5	60.9 (6.8) [4]	Coogan, Raubenheimer [52], Coogan (unpublished)
	Clover (late hyperphagia)				46.8 (8.9) [2]					Mattson, Barber [53]
	Clover (September)		17.3		22.4 [1]	15.3 (0.9) [3]	10.0 (0.8) [3]	65.4	60.4 [1]	Coogan, Raubenheimer [52], Coogan (unpublished)
	Clover White (late summer—fall, *Trifolium ripens giganteum*)	15.9 (4.2) [12]	34.2 (3.7) [12]			29.1(0.8) [12]	22.2	76.2	48.3 (4.2) [12]	Rode, Robbins [51]
	Cow parsnip (August)		11.4 (14.4)		17.5 (11.9) [4]	9.7 (6.9) [4]	5.1 (1.7) [4]	52.6	64.7 (10.3) [4]	Coogan, Raubenheimer [52], Coogan (unpublished)
	Dandelion (August)					9.7 (0.7) [3]	5.1 (0.6) [3]	52.6		Coogan, Raubenheimer [52], Coogan (unpublished)
	Graminoids (late hyperphagia, Sept. 01—den)				13.0 (8.0) [4]	9 (5.8) [4]	4.5	49.7	31.2 (4) [4]	Mattson, Barber [53]
	Grasses (late summer—fall, *Poa pratensis*, *Phleumpratense*, *Bromus gracilis*)	26.9 (3.1) [12]	21.9 (2.1) [12]			19.3 (1.4) [12]	13.5	70.2	57.1 (3.7) [12]	Rode, Robbins [51]
	Horsetails (August)					17.1 (4.3) [3]	11.6 (3.8) [3]	67.8		Coogan, Raubenheimer [52], Coogan (unpublished)
	Fireweed	30.9 (2.9) [2]	15.3^2^	4420 (39.5) [2]		9.05 (3.3) [2]	4.5^1^	49.9	61.9 (1.5) [2]	Fortin (unpublished)
	Fern-leaf lovage	23.1 (1.2) [5]	23.3^2^	4705 (243.6) [5]		17.1 (1.2) [5]	11.6^1^	67.8	56.1 (5.7) [5]	Fortin (unpublished)
	Bluegrass	44.6 (5.2) [2]	7.4^2^	4440 (12.7) [2]		10.5 (3.4) [2]	5.8^1^	55.0	67.5 (5.7) [2]	Fortin (unpublished)
**BERRIES**	Huckleberry (*Vaccinium membranaceum*)	14.6	72.5			3.7			20.7	Welch, Keay [54]
	Soapberry	18.0	70.3						22.3	Welch, Keay [54]
	Blueberries	17.9	63.8(0.1)	4472	67.2 (0.5)	5.6	1.1	18.9 (9.6)	24.3	Pritchard, Robbins [47]
	Crowberry (*Empetrum nigrum*)		35.7 (3.5)	4197 (400)	37.7 (2.9)	3.5.(0.1) [2]			47.2 (2.5) [2]	Coogan [62]
	*Vaccinum myrtilloides*		65.5 (3.1)	4077 (300)	62.4 (2.6)	4.7 (0.1) [4]	0.6 (0.2)	12.8	25. (1.3) [47]	Coogan [62]
	*Vaccinum scoparium*		68.8	4145	65.2	4.9 [1]	0.9	18.4	23.4 [1]	Coogan [62]
	*Vaccinum vitis-idaea*		68.1 (4)	4101 (160)	64.5 (3.3)	4.4 (0.3) [5]	0.4	9.1	23.9 (1.3) [5]	Coogan [62]
	Buffaloberry (*Shepherdia canadensis*)		66.4 (1.3)	4257 (295)	63.2 (1.1)	3.8				Coogan, Raubenheimer [52], Coogan [62]
	Berries (bearberry, strawberry, red twinberry, gooseberry, dwarf huckleberry, globe huckleberry, grouse whortleberry)	22.5 (7.1) [11]		4712 (88.1) [11]		6.0 (0.9) [11]			18.8 (2.2) [11]	Fortin (unpublished)
	Raspberry (*Rubus* spp.)	14.25 [14]		3649		8.4 [12]			45.6 [13]	USDA [56]
	Raspberry wild, Alaska (*Rubus* spp.)	15.5 [1]		3995		7.8 [1]			48.3 [1]	USDA [56]
	Cranberries raw (*Vaccinium macrocarpon*)	12.9 [4]		3574		0.39 [4]			35.7 [4]	USDA [56]
	Cranberries, wild Alaska (*Viburnum edule*)	14.0 [1]		3928		1.1 [1]			47.9 [1]	USDA [56]
	Gooseberries, raw (*Ribes* spp)	12.1 [15]		3627		0.88 [2]			35.4	USDA [56]
	Blackberries, raw (*Rubus* spp)	11.9 [5]		3629		1.39 [5]			44.7 [4]	USDA [56]
	Blackberries, wild (*Rubus* spp)	11.9 [3]		4351		0.84 [3]			26.7 [2]	USDA [56]
	Blueberries, raw (*Vaccinium* spp.)	15.8 [12]		3609		0.74 [12]			15.2 [4]	USDA [56]
**ROOTS**	Tubers (carrots-yams)	16.8	57.8 (1.8)	4123	57.6 (2.6)	8.3	4.4	52.7 (6.1)	18.1	Pritchard, Robbins [47]^3^
	*Hedysarum alpinum*		41.4 (1.9) [16]	3720 (330) [16]	42.4 (1.5) [16]	12.6 (1.8) [16]	7.6^1^ (0.4) [16]	60.3	43.1 (1.3) [16]	Coogan [62]^a^
	*Hedysarum alpinum*		33.7 (1.9) [51]	4204 (430) [21]	36.0 (1.6) [51]	15.7 (0.2) (117)	10.3^1^ (0.2) (117)	65.6	48.7 (1.4) [51]	Coogan [62]^b^
	*Hedysarum alpinum*					12.5	7.6^1^	60.42		Hamer, Herrero [57]
	Yellow Hedysarum					10.75	6.0^1^	55.9		Hamer, Herrero [57]
	Pocket gopher cache				47.9 (18.6) [10]	8.6 (2.9) [8]	4.1	47.9	10.8 (3.6) [8]	Mattson, Barber [53]
	Vole cache				71.5 [1]	5.1 [1]	1.0^1^	20.3	5.2 [1]	Mattson, Barber [53]
	Pondweed root				68.4 (8.6) [5]	8.7 (0.6) [2]	4.2^1^	48.3	6.4 (0.7) [2]	Mattson, Barber [53]
	Yampa root				71.8 (8) [57]	5.9 (1.7) [26]	1.7^1^	29.5	6.5 (2.7) [27]	Mattson, Barber [53]
	Sweet-Cicely root				63.6 (14.2) [11]	7.8 (1.5) [4]	3.4^1^	43.7	13.2 (5) [4]	Mattson, Barber [53]
	Biscuitroot				63.3 (8.7) [69]	5.2 (1.4) [35]	1.1^1^	21.6	9.5 (2.5) [34]	Mattson, Barber [53]
	Carrot (*Daucus carota*)	11.7 [33]		3501		7.9 [22]				USDA [56]
	Yam (*Dioscorea* spp.)	30.4 [12]		3882						USDA [56]
	Sweet potato (*Ipomoea batatas*)	22.7 [7]		3785						USDA [56]
	Elk thisle	13.8 (1.6) [3]		3942 (76) [3]		13.5 (2.5) [3]	8.4^1^	62.5	48.0 (9) [3]	Fortin (unpublished)
	Glacier lily	13.8 (2.7) [2]		4124 (81) [2]		8.5 (1.6) [2]	4.0^1^	47.4	22.9 (8) [2]	Fortin (unpublished)
	Licorice root	28.0 (3.8) [2]		3827 (470) [2]		6.6 (0.6) [2]	2.4^1^	35.7	36.3 (6.7) [2]	Fortin (unpublished)
	Yampa root	38.7 (10.4) [6]		3729 (132) [6]		5.2 (1.9) [6]	1.1^1^	21.6	28.7 (8.3) [6]	Fortin (unpublished)
**ANTS**	*Camponotus herculeanus*	31.2	88.4	2190	20.6	43.8	34.5^1^	78.7	9.3	Swenson, Jansson [60]
	*Formica* spp	26.7	77.1	2080	17.7	55.9	46.7^1^	83.5	17.4	Swenson, Jansson [60]
	*Formica* spp (pupae)	24.4	64.3	2370	17.8	54.9	45.7^1^	83.2	11.3	Swenson, Jansson [60]
	*Acanthomyops* spp (worker)			5300		31.0 (5.8)	21.5^1^	69.4		Noyce, Kannowski [59]
	*Acanthomyops* spp (pupae)			6000		40.1 (3.8)	30.7^1^	76.6		Noyce, Kannowski [59]
	*Camponotus* spp (workers)			4800		34.5	25.1^1^	72.7		Noyce, Kannowski [59]
	*Camponotus* spp (aletes)			6500		26.4	16.9^1^	64.0		Noyce, Kannowski [59]
	*Formica* spp. (workers)			4400		27.0	17.5^1^	64.8		Noyce, Kannowski [59]
	*Formica aserva* (larvae)			5260 (860) [2]		55.9 (8.5) [2]	46.7^1^	83.5		Coogan [62]
	*Formica aserva* (ants)			4730 [2]		50.7 (4.3) [2]	41.4^1^	81.7		Coogan (unpublished)
	*Formica ulkei* (larvae)			6360 (700) [2]		66.8 (6.9) [2]	57.7^1^	86.4		Coogan [62]
	*Formica ulkei* (ants)			6600 [1]		69.1 [1]	60.0^1^	86.9		Coogan (unpublished)
**TERRESTRIAL MEATS**	Deer	26.1	93.0 (1.3)	7316	94.6 (0.7)	45.1	40.4	89.5 (1.5)	6.3	Pritchard and Robbins [47]
	Beef	37.0	93.3 (1.6)	6748	96.5 (0.7)	53.0	50.7	95.7 (0.8)	4.7	Pritchard and Robbins [47]
	Ground squirrels	28.8	76.1^3^ (1.5)	5284	84.5^3^ (0.8)	67.5	57.7	85.5^3^ (2.2)	17.3	Pritchard and Robbins [47]^3^
	Wapiti (spring and estrus)				90.0	80.0	71.0^1^	88.8		Mattson, Barber [53]
	Wapiti (early hyperphagia)				92.0	62.0	52.9^1^	85.2		Mattson, Barber [53]
	Wapiti (late hyperphagia)				93.0	45.0	35.7^1^	79.3		Mattson, Barber [53]
	Bison and moose (spring and estrus)				92.0	81.0	72.0^1^	88.9		Mattson, Barber [53]
	Bison and moose (early hyperphagia)				94.0	67.0	57.9^1^	86.4		Mattson, Barber [53]
	Bison and moose (late hyperphagia)				96.0	53.0	43.8^1^	82.6		Mattson, Barber [53]
	Bison	23.1		5691		86.5	77.6^1^	89.7		Fortin (unpublished)
	Mule deer	25.7		5656		86.6	77.7^1^	89.7		Fortin (unpublished)
	Elk, game meat, raw (*Cervus elepahyus*)	25.6 [30]		4333		89.6 [22]	80.7^1^	90.1		USDA [56]
	Caribou, game meat, raw (*Rangifer* spp.)	28.6 [45]		4448		79.3 [30]	70.3^1^	88.7		USDA [56]
	Moose, game meat, raw (*Alces alces*)	24.5 [36]		4172		91.0 [35]	82.1^1^	90.3		USDA [56]
	Deer, game meat, raw (*Odocoileus* spp)	26.4 [44]		4540		86.9 [33]	78.0^1^	89.8		USDA [56]
	Squirrel, game meat raw (Sciuridae)	26.2 [17]		4585		81.1 [17]	72.2^1^	89.0		USDA [56]
	Rabbit, game meat, raw (*Sylvilagus* spp., *Oryctolagus* spp.)	25.5 [31]		4472		85.5 [32]	76.6^1^	89.6		USDA [56]
**PINE NUTS**	*Pine* spp	97.7 [8]		6887		14.0 [8]	4.4^1^	31.3	3.8 [5]	USDA [56]
	*Pinus edulis*	94.1 [1]		6684		12.3 [1]	2.6^1^	21.53	11.4 [5]	USDA [56]
	*Pinus edulis*	95.0	41.2^3^ (0.9)	6484	50.1^3^ (3.1)	8.8	5.0^3^	57.2 (3.1)	40.3	Pritchard, Robbins [47]
	Confier seeds			7000						Fogel, Trappe [61]
	Whitebark pine nut		66.5		49.2	12.8	3.2	24.7	34.8	Mattson, Barber [53]
	Whitebark pine nut			6111 [1]		14.8 [1]	9.6 [1]	64.9		Coogan [62]
	Whitebark pine nut	86.1 (2.8) [2]	21.1	5764 (94) [2]		11.6 (1.9) [2]	1.9	16.8	80.2 (3.6) [2]	Fortin (unpublished)
**TROUT**	Cutthroat trout	25.0	89.8 (1.1)	5715	94.5 (1.1)	69.6	66.3	95.2 (0.6)	11.1	Pritchard, Robbins [47]
	Trout	29.5 (4.6) [12]		6258 (299) [6]		65.4 (8.1) [12]	62.2^1^	95.2		Fortin (unpublished)
	Trout, rainbow, wild, raw *(Salmo gairdneri* Richardson)	28.1 [30]		4230		72.8 [28]	63.7^1^	87.6		USDA [56]
	Trout, rainbow, farmed, raw (*Salmo gairdneri*, Richardson)	26.2 [8]		5382		76.1 [8]	67.1^1^	88.2		USDA [56]
**FALSE-TRUFFLES**	*Rhizopogon* spp	34.5 (11.5) [6]		4884 (141) [6]		11.0 (2.4) [6]			52.1 (11.2) [6]	Fortin (unpublished)
	Mushroom (Basidiocarp)		83.5^2^		51.1 (4.5) [4]	17.8 (5.1) [4]			12.6 (2.8) [4]	Mattson, Barber [53]
	Fungi		76–83^2^			15–35			13–18	PC from Mealey [63], TDF from Cheung [64]
	Fungi	70–94 (average = 84)		4500 (1.2)		26.1 (10.4)				Fogel, Trappe [61]

1: PDig was estimated using the relations presented in Pritchard and Robbins [47].

2: DMDig was estimated using the relations presented in Pritchard and Robbins [47]

3: DMDig, EDig and PDig (%) were measured on black bears (*Ursus americanus*).

a: Independent laboratory.

b: Annual average values. University of Alberta laboratory.

DM (%) = Dry matter (% of fresh matter); DMDig (%) = Digestible dry matter; GrossE (kcal/kg) = Gross energy; EDig (%) = Energy digestibility; PC (%) = Protein content; PDig (g) = protein digested per 100 gr of protein; PDig (%) = Protein digestibility; TDF (%) = Total dietary fiber. DMDig, GrossE, EDig, PC and PDig in a dry matter basis. When information was available standard deviation is included in parenthesis and sample size in brackets.

The roots category included 15 species (Table 3). For the Alberta and FR ecosystem, we used nutritional estimates for one root species: alpine sweetvetch (*Hedysaraum alpinum*; [55]). For the GYE we used all root species to estimate the average and SD of nutritional parameters. To estimate the dry matter content and gross energy, we used values obtained from GYE and USDA National Nutrient Database for carrot (*Daucus carota*), yam (*Dioscorea* spp.), and sweet potato (*Ipomoea batatas*).

The pine nuts category included whitebark pine and other conifer seeds (piñion pine *Pinus edulis*) (Table 3). Nutritional information for the berries category was obtained from six common species in Alberta, Flathead and GYE (Table 3). For ants, nutritional information included values for workers and pupae (Table 3).

In the terrestrial meats category, we included ungulates and rodents (Table 3). Ungulate nutritional values were based on an average for deer (*Odocoileus* spp.), elk (*Cervus elaphus*), bison (*Bison bison*) and moose (*Alces alces*) (Table 3). Nutritional information for false-truffles corresponds to *Rhizopogon* spp and basidocarp (Table 3). Trout category included cutthroat trout and values from GYE and from USDA National Nutrient Database for wild and raw trout (*Salmo gairdneri*, Richardson; Table 3).

Miscellaneous food category reported in Munro, Nielsen [50], and garbage and debris categories reported in Mattson, Blanchard [30], were not considered in our analysis because these food items were not explicitly identified, their nutritional information was not available, and their contributions to overall bear diets were minimal. The exclusion of the miscellaneous food category is not likely to significantly affect our conclusions.

### Nutritional values per food categories

Nutritional information included six components: dry matter (%, DM); dry matter digestibility (%, DMDig); gross energy (kcal/kg, GrossE); energy digestibility (%, EDig); crude protein (%, PC); and protein digestibility (%, PDig). All components, except DM are expressed on a dry matter basis. Nutritional information for each category was estimated using previous published and some unpublished data (Table 3). Because the number of samples in some food categories were small and, thereby, precluded an estimate of variation, we assumed in those cases a standard deviation equal to 10% of the average reported nutritional value.

### Model structure

We used Stella 10.2 [65] to build a dynamic model that estimates the digestible energy and protein in one kilogram of fresh bear diet using the food habits and nutritional information described above. Stella is a programing software that uses icons as an interface and it is specialized for dynamic modelling [66, 67]. This software organizes different variables and parameters of a process or system depending on their functional relationships. The interactions among components occur through time taking into account the previous state of the components (dynamic modelling). This allows the user to simulate ecological processes and observe how variables evolve through time.

The model assesses the digestible energy and protein in one kilogram of fresh diet (i.e. as fed or wet weight basis), in a daily time step, where day one corresponds to April 15, and the final day corresponds to October 15, for a total of 183 days. There were three model inputs: 1) the fecal volume per food item, which was obtained from food habits information. Because this data came in bi-weekly periods, the model interpolates between these values to obtain the fecal volume per day; 2) the CFs, which were fixed or variable depending on the model analysis that we ran (see Correction factors analysis; Table 1); and, 3) the nutritional estimates (i.e. DM, DMDig, GrossE, EDig, PC, PDig) for each food category. These values were obtained randomly from a normal distribution curve. This curve was estimated from the average and standard deviations presented in Table 2. When values were negative we assumed a value of zero. Due to the variability in nutritional values, one thousand repetitions were run per simulated scenario.

Model outputs included daily digestible energy and protein content (fresh diet base). Digestible energy and protein contributions per food category were also estimated to identify the foods that most contributed energy and protein. Results were reported on a “per kilogram of fresh diet” rather than “dry matter” basis because it simplifies future estimations of foods requirements (kg) and potential daily food intake.

### Model calculations

The model runs in three consecutive calculations (sections) as described below. A numeric example is presented in the S1 File.

### First section: from fecal volume (%) to Digestible Dry Matter (DMDig)

The model uses the fecal volume per food item (%.FV(fi)) and its corresponding CFs to estimate the grams of digestible dry matter per food item (g. DMDig(fi)). Calculations follow the same steps presented on Hewitt and Robbins [46]. %.FV(fi) is multiplied by their corresponding CFs (CF(fi)) and adjusted to the total fecal volume in the diet (Eq 1).

$$\text{g}.\text{DMDig}(\text{fi})=\%.\text{FV}(\text{fi})\times \text{CF}(\text{fi})÷\sum_{\text{fi}=1}^{\text{n}}\left(\%.\text{FV}(\text{fi}\right)\times \text{CF}(\text{fi}))\times 100$$ (1)

We used the fecal volumetric results presented in McLellan and Hovey [49], Mattson, Blanchard [30] and the raw data from Fortin, Schwartz [33]. For Munro, Nielsen [50] we estimated FV from the DMDig information presented. For the ecosystems diet analysis we used fixed CFs (Table 1) based on those values previously published in Fortin, Schwartz [33]. To explore the impact of CFs on our results we allowed the CFs to vary (see Correction factors analysis). At the end of this first section, food items were grouped into the different food categories.

### Second section: from Digestible Dry Matter intake (DMDig) to Fresh Food Intake

The model estimates the grams (g) of each food category (f) in one kilogram of fresh diet (g.FFDiet(f)). To transform the digestible dry matter per food category (g. DMDig(f)) to grams of fresh food (g.FFood(f)), the g.DMDig(f) is divided by their corresponding dry matter digestibility (%.DMDig(f)) and dry matter content (%.DM(f)) (Eq 2). DM and DMDig are obtained randomly from a normal distribution curve using data in Table 2. Grams of each food item in the fresh diet base is obtained by dividing the g.FFood(f) by the sum of all food items and multiplying by 1000 (g) (Eq 3).

g.FFood(f)=g.DMDig(f)÷(%.DM(f)×%.DMDig(f))×10000 (2)

$$\text{g}.\text{FFDiet}(\text{f})=\text{g}.\text{FFood}(\text{f})÷\sum_{\text{f}=1}^{\text{n}}\text{g}.\text{FFood}(\text{f})\times 1000$$ (3)

### Third section: estimations of digestible energy and digestible protein in one kilogram of fresh diet

In the second phase, the model uses the g.FFdiet (f) and the nutritional values (Table 2) to estimate the contribution of digestible energy and protein per food category and later adds these contributions to obtain the total digestible energy and protein in one kilogram of fresh diet.

Digestible energy per food category (kcal.DigestibleE(f)) is the product of g.FFDiet(f), dry matter content (%.DM(f)), gross energy (kcal/g.GrossE(f)) and energy digestibility of each food category (%.EDig(f)). DM, GrossE and EDig are obtained randomly from a normal distribution curve using data in Table 2 (Eq 4). Digestible energy for the total diet (kcal. DigestibleE (diet)) is the sum of the digestible energy per food category (Eq 5).

kcal.DigestibleE(f)=g.FFDiet(f)×%.DM(f)×kcal/g.GrossE(f)×%.EDig(f)÷10000 (4)

$$\text{kcal}.\text{DigestibleE}(\text{diet})=\sum_{\text{f}=1}^{\text{n}}\text{kcal}.\text{EDig}(\text{f})$$ (5)

Digestible protein per food category is the product of the g.FFdiet(f), dry matter content (%.DM(f)), protein content (%.PC(f)), and protein digestibility (%.PDig(f)) of each food category. PC, PDig are obtained randomly from a normal distribution curve using data in Table 2 (Eq 6). Digestible protein for the total diet (g.DigestibleP (diet)) is the sum of the digestible protein per food category (Eq 7).

g.DigestibleP(f)=g.FFDiet(f)×%.DM(f)×%.PC(f)×%.PDig(f)÷1000000 (6)

$$\text{g}.\text{DigestibleP}(\text{diet})=\sum_{\text{f}=1}^{\text{n}}\text{g}.\text{DigestibleP}(\text{f})$$ (7)

Because the model estimates each nutritional value randomly from a normal distribution, we ran 1000 repetitions. Averages and standard deviations (SD) were estimated.

### Correction factors analysis

Estimates of bear food habits derived from fecal analysis are dependent on the CFs applied, which therefore have a direct influence on our assessment of of nutritional parameters on bear diets. Therefore, we developed two analyses to explore the impact of using different CFs on our results. First, we ran our original model allowing CFs to vary within their range (Table 1) while all other settings were kept as previously described. Our second analysis focused on the influence of ungulate CFs (CFungulate) on our estimates of nutritional parameters of bear diets. Here we designed a simpler analysis, in which we simulated four diets composed of ungulates and four other common food items: green vegetation; roots; fruit; and pine nuts. In these simpler models we varied the proportion of ungulate in fecal volumetric analysis from 1 to 100% and varied the ungulate CFs from 1 to 12 to evaluate changes in the contribution of energy from the terrestrial meat category.

## Results

### Digestible energy and protein per food item (fresh food base)

**Fig 1:**
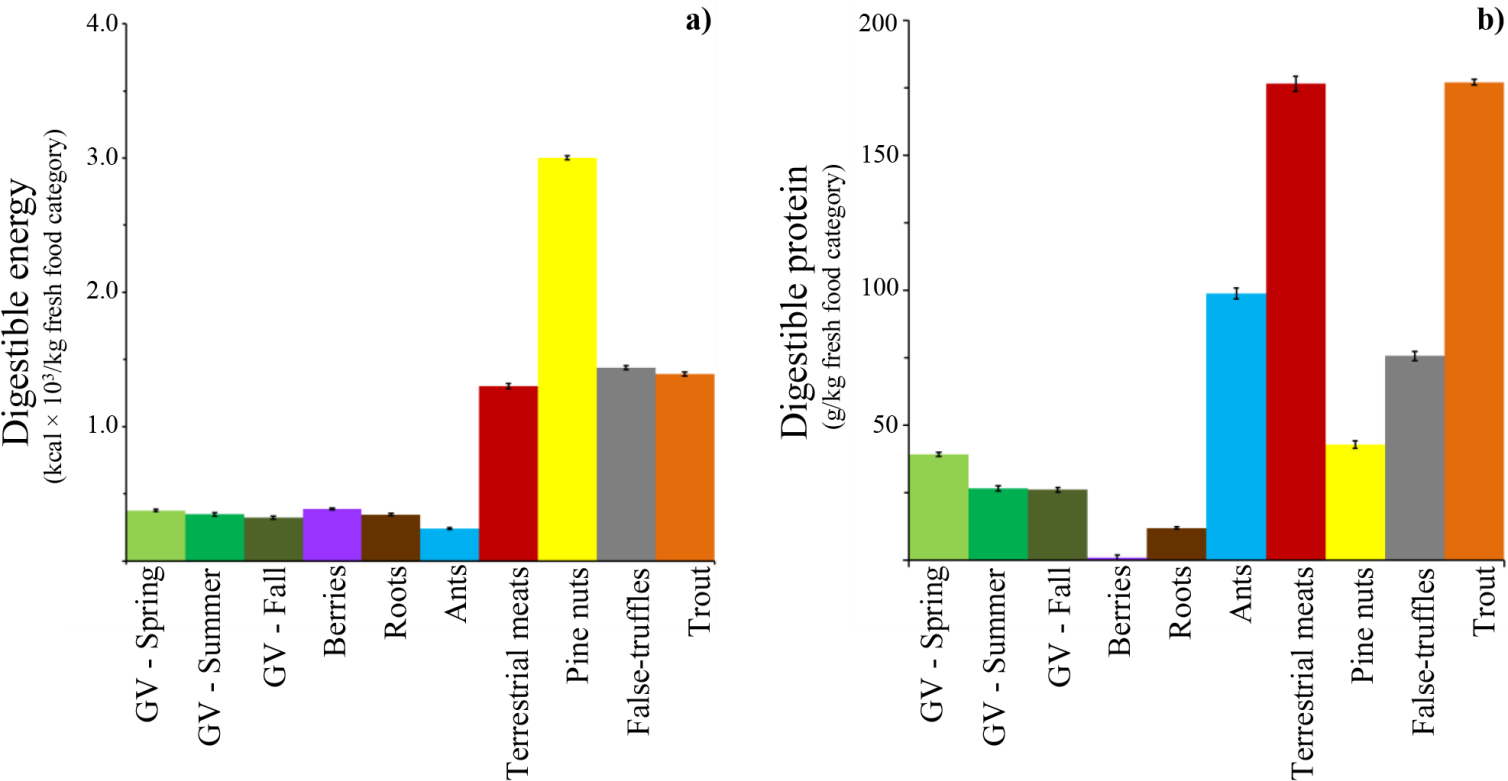
(a) Digestible energy (kcal/kg fresh food) and (b) digestible protein (g/kg fresh food) per brown bear food item category. Error bars indicate standard error (n = 1000 repetitions). Digestible energy and protein were estimated using the nutritional values of each food category. Nutritional values were obtained randomly for a normal distribution curve built with the average and SD presented in Table 2.

As expected, digestible energy and protein (g/kg fresh food) was noticeably different between food categories (Fig 1). Plant matter had lower levels of digestible energy and protein than animal matter, pine nuts and false-truffles. Pine nuts have the highest level of digestible energy because of their very low water content and high fat content, followed by false-truffles, terrestrial meats and trout (Fig 1A). Digestible energy in one kilogram of green vegetation, berries or roots are ~1/8 that in nuts and ~1/4 that in terrestrial meats (Fig 1A). Digestible protein was higher in trout and terrestrial meat than false-truffles and ants. Digestible protein in one kilogram of terrestrial meats or trout is ~15 times higher than in one kilogram of roots, and ~5 times higher than in one kilogram of green vegetation (Fig 1B).

### Digestible energy in bear diets

**Fig 2:**
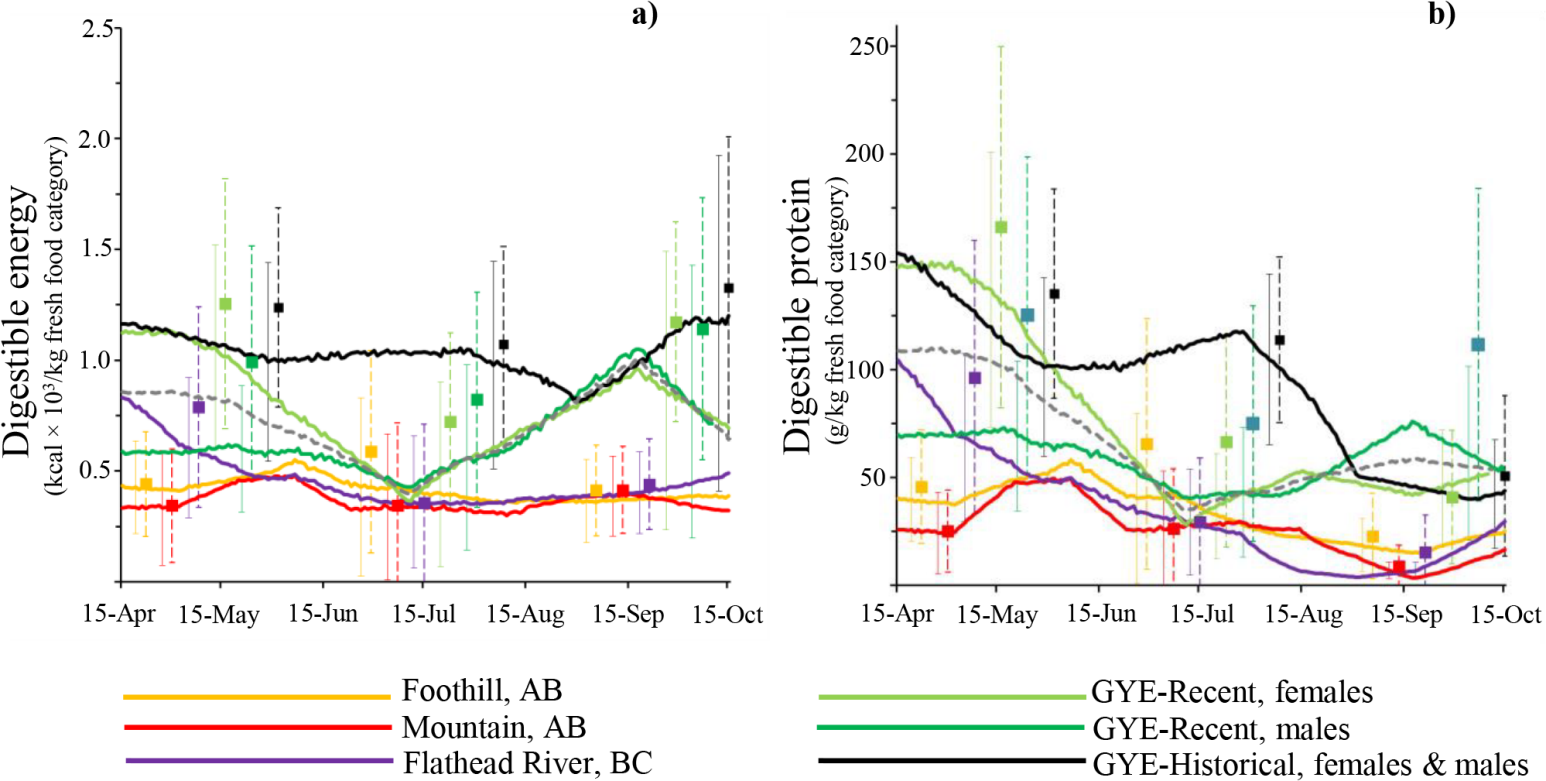
(a) Digestible energy (kcal) and (b) digestible protein (g) in one kilogram fresh brown bear diet across different ecosystems. Ecosystem diets include the “Foothills” and “Mountains” of west-central Alberta (Canada), “Flathead” river drainage in southeast British Columbia (Canada) and the Greater Yellowstone Ecosystem (GYE, USA). For the GYE, we present the recent diets for both female (“GYE-Recent, female”) and male (“GYE-Recent, male”), the average recent diet (“GYE-Recent”), and the historical diet “GYE-Historical, females & males” diets. Digestible energy and protein were estimated based on the proportion of digestible dry matter intake obtained from food habit studies in these ecosystems [30, 33, 49, 50], fixed correction factors (CFs) are presented in Table 1, and nutritional information. Nutritional values were obtained randomly for a normal distribution curve estimated from the average and SD presented in Table 2. Continues bars indicate ±1.96×SD (n = 1000 repetitions). Squares and dashed bars represent the results (average and ±1.96×SD) when CFs were allowed to vary (Table 1).

Estimated digestible energy varied through the season in all ecosystems (Fig 2A). All bear diets in the GYE had the highest levels of digestible energy, although the historical diet had the highest level throughout the three seasons. The recent GYE diets displayed two distinct peaks in digestible energy content: one in spring (until 15^th^ May), and other in late summer (31^th^ August to 30^th^ Sept). During spring, recent male GYE diets had a digestible energy content ~50% lower than the recent female and historical GYE diets. The FR diet had a digestible energy level in spring that was similar to the recent male diet in GYE, but later decline to have similar values of the Alberta bear diets. Bear diets in western Alberta had the lowest levels of digestible energy in all three seasons. These diets showed a small peak of digestible energy during early summer (15^th^ of May to 30^th^ of June). In early spring, digestible energy in the diets of Alberta bears were ~1/3 of those in the FR. During late summer and early autumn, recent diets in GYE provided ~2 times more digestible energy than in the FR and the Foothills and Mountains in western Alberta.

### Digestible protein in bear diets

Digestible protein varied through the seasons for all ecosystems (Fig 2B) and was highest in the spring and early summer. Historical diet in the GYE provided one of the highest levels of digestible protein throughout the three seasons, which was ~3 times higher in the summer than the recent diets in the GYE (Fig 2B). During spring, the male GYE diet had a digestible protein content ~50% lower than the female and historical GYE diets. The FR diet had protein levels higher than the recent male diet in GYE during early spring, but in summer and fall protein levels decreased in the FR to less than ~50% of the diets in the GYE. Diets in Alberta have the lowest levels of digestible protein through the entire season. Digestible protein in Alberta Mountain bear diet was ~1/4 that of the recent GYE female diet during spring and early summer.

### Energy contribution per food item

**Fig 3:**
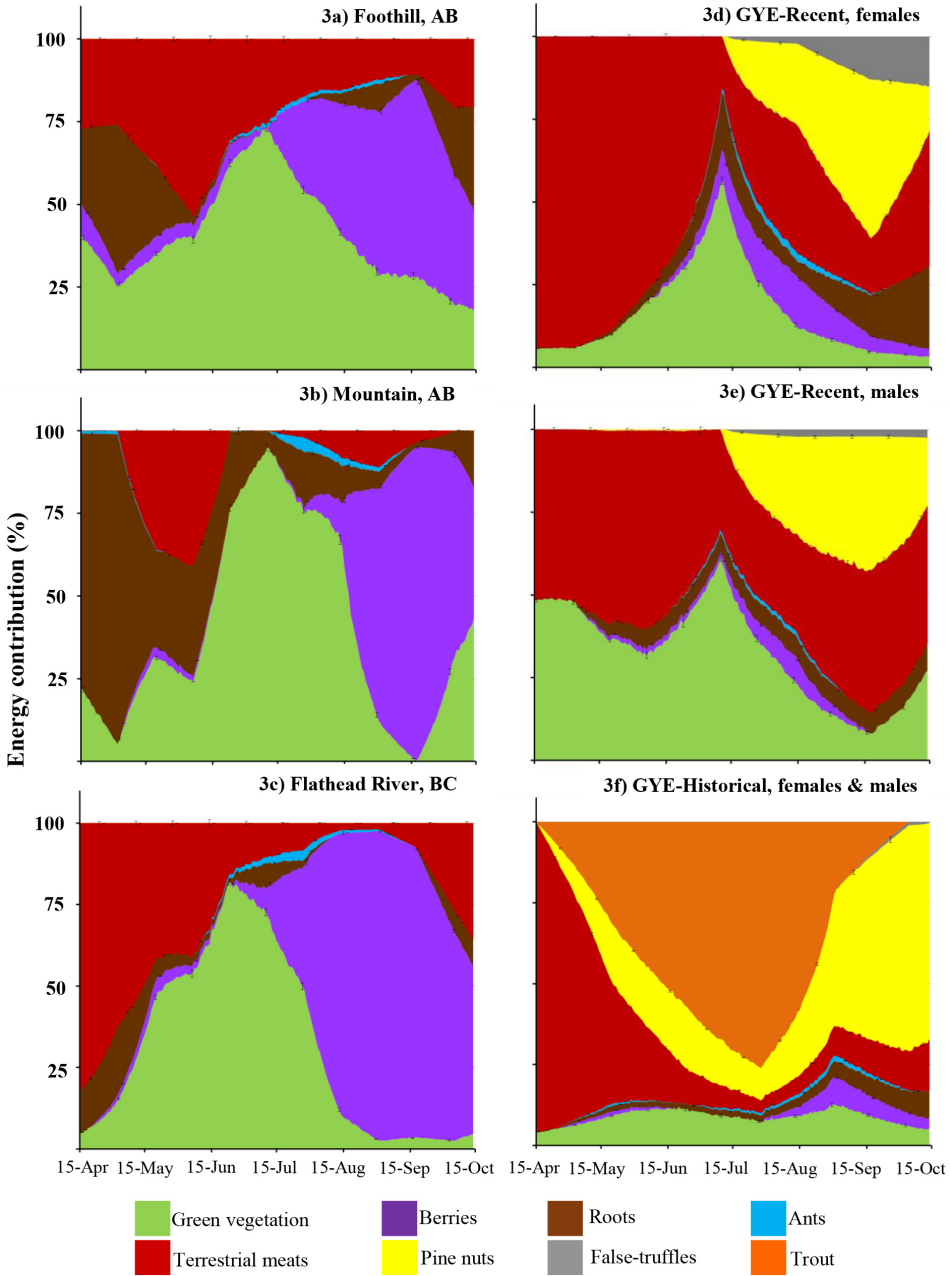
Percent digestible energy contribution per food item category (fresh diet base) across ecosystems. Contribution was estimated based on the total digestible energy in one kilogram of fresh diet. Ecosystem diets include: (a) Foothills and (b) Mountains of west-central Alberta (Canada), (c) Flathead River drainage in British Columbia (Canada) and the Greater Yellowstone Ecosystem (GYE, USA). For the GYE, we present the recent diets for both (d) female “GYE-Recent, female”, (e) male “GYE-Recent, male”, and the (f) historical diet “GYE-Historical, females & males” diets. Continues bars indicate ±1.96×SD (n = 1000 repetitions).

Consumption of terrestrial meats was the primary reason for the higher digestible energy occurring during spring in the FR and GYE bear diets (Fig 3C, 3D, 3E and 3F). During summer and early fall, high energy levels in the historical GYE diets were due to consumption of trout and pine nuts. In the recent GYE diets, the dietary proportion of pine nuts decreased, which increased the relative importance of terrestrial meats, green vegetation and false-truffles (Fig 3D, 3E and 3F). In the recent period, GYE bears required ~4 kg of green vegetation to supply the same amount of digestible energy as one kilogram of trout that was historically available. Similarly, GYE bears needed ~3 kg of terrestrial meat or ~8 kg of green vegetation to supply the same amount of digestible energy as one kilogram of pine nuts.

Berries were an important source of dietary energy for FR and western Alberta bears during summer and early fall. Despite this, the digestible energy content of their diets was lower than that in the GYE diets. Bears in these ecosystems need to consume ~3.4 or ~7.8 kg of berries to obtain the same amount of energy as one kilogram of terrestrial meat or pine nuts in the GYE, respectively.

In the FR ecosystem, digestible energy during spring is derived primarily from green vegetation and roots, while terrestrial meats provide only 25% of digestible energy (Fig 3A). In the Mountain ecosystem, the energy contribution during spring is primarily from roots (Fig 3B). During late summer and fall digestible energy is mainly derived from green vegetation and berries (Fig 3A and 3B). The absence of high energy foods in the Alberta diets, such us terrestrial meats and pine nuts, may restrict the capacity of the individuals to meet their energy demands and accumulate fat late in the season.

### Protein contribution per food item

**Fig 4:**
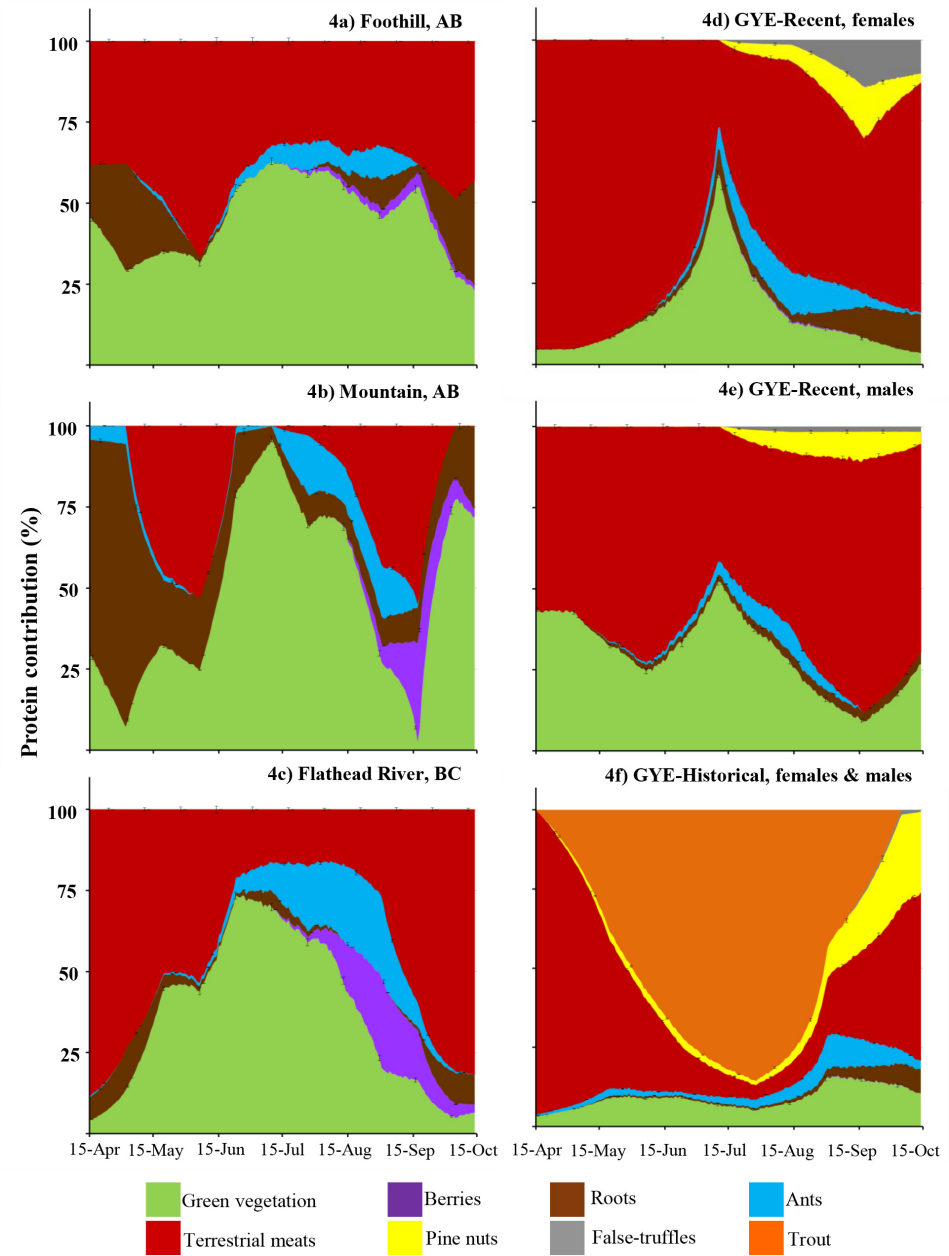
Percentage of digestible protein contributed per food item category (fresh diet base) across ecosystems. Contribution was estimated based on the total digestible protein in in one kilogram of fresh diet. Ecosystem diets include: (a) Foothills and (b) Mountains of west-central Alberta (Canada), (c) Flathead River drainage in British Columbia (Canada) and the Greater Yellowstone Ecosystem (GYE, USA). For the GYE, we present the recent diets for both (d) female (“GYE- Recent, female”), (e) male (“GYE- Recent, male”), and the (f) historical diet “GYE-Historical, females & males”” diets. Continues bars indicate ±1.96×SD (n = 1000 repetitions).

High protein levels during spring in the FR and GYE diets (Fig 4B) are due to the consumption of terrestrial meats, which provide more than 50% of the total protein (Fig 4C, 4D, 4E and 4F). In the FR ecosystem, terrestrial meat was an important source of digestible protein throughout the year. Historical diets in the GYE had the highest protein levels during summer and early fall due to the consumption of trout. Lower digestible protein in the recent male and female GYE diets was due to the decrease of terrestrial meat and trout consumption, which was replaced by green vegetation. Bears in the GYE required ~6 kg of green vegetation to supply the same amount of digestible protein as one kilogram of terrestrial meat or trout.

In the Foothill ecosystem green vegetation and terrestrial meat (~30%) were the main source of digestible protein in spring (Fig 4A). In the Mountain ecosystem, roots were the main source of digestible protein (> 75%) with the rest coming mainly from green vegetation (Fig 4B). Low protein or low energy foods during spring, such as roots and green vegetation (Fig 1B), may restrict lean mass growth and milk production and thus affect reproductive success of adult females. For example, bears in Alberta need to consume ~15 kg of roots or ~5kg of green vegetation to obtain the same amount of digestible protein as in one kilogram of terrestrial meat.

### Correction factors

Variable CFs created noticeably different diet estimates than when we used a fixed CF (Fig 2). Digestible energy and protein were generally higher when we allowed the CFs to vary during model runs as compared to choosing more conservative CFs (Fig 2A and 2B). This increase in protein and energy estimates was most noticeable when terrestrial meat was an important component of bear diets (Figs 2, 3 and 4).

**Fig 5:**
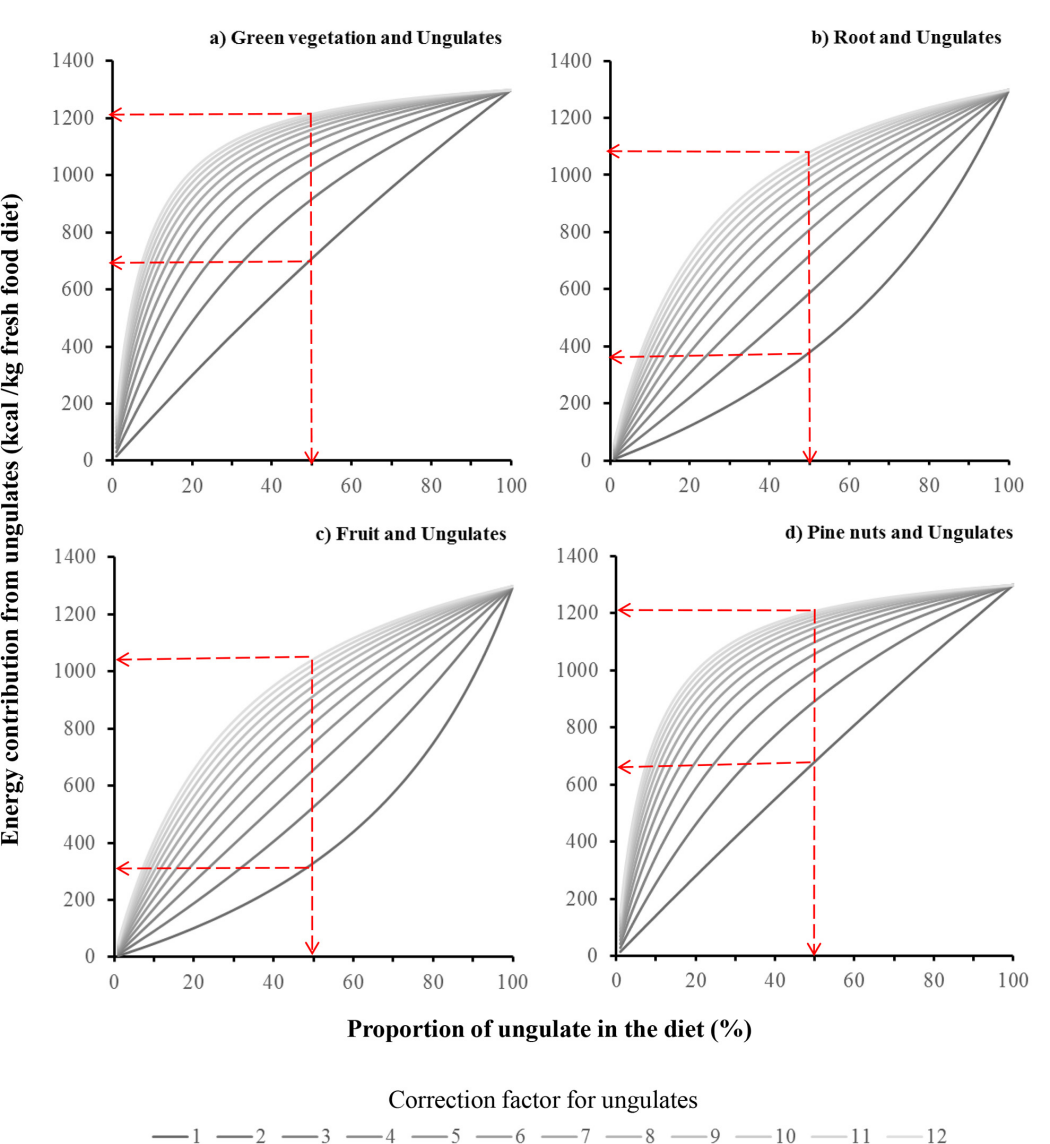
Energy contribution from terrestrial meat on bear diets under different CFs for ungulates and proportion of ungulates on the diet. We simulated four diets composed of ungulates and four other common food items: green vegetation; roots; fruit; and pine nuts. Red lines show the energy contribution from ungulates for a diet with 50% of ungulates and 50% of a) green vegetation; b) roots; c) fruit or d) pine nuts.

As expected, the energy contribution from ungulates increased as the CFungulate and proportion of ungulates in the diet increased. This energy increase followed a logarithmic growth shape in most scenarios depending on the CFungulate used and the nutritional characteristics of other food items (Fig 5). When CFungulate was <6, the differences in energy contribution were higher, suggesting that using CFungulate for terrestrial meat below this threshold will have a stronger impact on dietary estimates (Fig 5).

Clearly, CFs used for terrestrial meat have an important influence on the assessment of the nutritional parameters of bear diets. This is a consequence of two related factors: 1) the large variability in CFs used for terrestrial meat that depends on the amount of hair and skin consumed in addition to the meat; and 2) the high energy and protein content of terrestrial meat compared with most other food categories (Fig 1). Because of these interactions, the potential error created by choosing incorrect CFs increases as meat availability increases across the ecosystems. Therefore, in our study we adopted a conservative approach and used a fixed CF of 3 for ungulates and 4 for small mammals. These low values provide conservative estimates of the importance of meat in the diet, and therefore the relative importance of meat in providing digestible energy and protein in our analyses might be underestimated in all diets.

## Discussion

Bear diets differ in the patterns of digestible protein and energy across ecosystems and seasons. These patterns can be associated with differences in body size and population density between ecosystems. Digestible energy and protein of bear diets were highest in the GYE, followed by FR and Alberta ecosystems. Ecosystems in Alberta, particularly the Mountains, had the lowest levels of digestible energy and protein through all seasons. This is consistent with the low reproductive rates observed in Banff National Park [68] and low bear densities in the Mountain and Foothill ecosystems [20]. In these less productive ecosystems, plant-based foods, such as roots and green vegetation, are an important source of protein and energy in spring and fall, but those foods are not as energy or protein dense as animal matter. There are other nutritional aspects of Alberta ecosystems that might also contribute towards low observed bear’s densities. Alberta ecosystems have a shorter growing season and, therefore, a shorter amount of time for bear foraging [50]. While habitat disturbances (e.g. logging, energy development, and road building) may increase the production of berries, green vegetation and roots in new open areas, these activities may increase human-bear conflicts and therefore increase bear mortalities [69–72].

The role of dietary protein intake on individual body size and population productivity of bears is controversial. Meat-rich diets have been correlated with increased bear body size and population density [73]; but when populations without access to salmon are excluded from the comparison, there is only a weak relationship between the proportion of terrestrial meat in the diet and average body size [11] and a negative relationship with population density [18]. Bears in the FR have one of the smallest body sizes among North American brown bear populations but one of the highest population densities among interior bear populations [3, 18]. Our results showed that diets in the FR ecosystem had protein levels similar to the recent-average diet in the GYE during spring, but energy levels were not as high as in the GYE diets during late summer and fall. A rich protein diet in spring may improve lean mass accumulation and milk production for lactating females, which may enhance reproductive success [4, 14, 16]. For example, brown bear populations in northern Sweden were able to maintain or gain mass in spring when compared with southern populations with the authors suggesting that this may be due to more abundant sources of protein in the northern ecosystems [74]. In the FR ecosystem black bears (*Ursus americanus*) were also able to gain weight during the spring [18]. High population density in FR has been attributed to their smaller body size which reduces nutrients needs, and the presence of abundant fall berries providing sufficient energy for fattening prior to hibernation [18]. Our results suggest that protein intake in the FR, especially early in the season, maybe more important than previously thought.

Recent research highlights the importance of non-protein macronutrients (lipid and carbohydrate) to brown bear fitness [52, 75], and behavior aimed at acquiring specific ratios of protein, carbohydrate and lipid may confound energy-based foraging models [76–78]. Indeed, captive and wild grizzly bears have shown the ability to balance their intake of protein and non-protein macronutrients in proportions that optimize energy intake and maximize mass gain [75, 79]. Diets imbalanced in macronutrients have associated costs. For example, high protein diets increase maintenance cost and decrease the efficiency of mass gain [75, 79, 80]. Therefore, diets higher in protein or energy are not necessarily better, nor do they capture all aspects of diet quality. However, complete macronutrient estimates (and their digestibility) of foods available to grizzly bears across these ecosystems are not to our knowledge available [52], which precludes a macronutrient specific approach. As such, our results should be interpreted with these limitations in mind; however, our protein- and energy-based approach is both informative and appropriate under these circumstances.

Major differences in the protein and energy in bear diets across ecosystems were largely due to the presence or absence of a few highly nutritious food items, such as terrestrial meat (mainly ungulates), pine nuts or trout. As a consequence, small changes in consumption of nutritious foods can have large impacts on bear nutrition. This pattern was observed when comparing the historical [30] and recent bear diets in the GYE [33]. However, individual capacity to switch between foods is constrained by factors that were not measured in this study, such as food abundance and distribution, bear social structure, and bear physiology (e.g. digestion rate, stomach capacity, and nutrient preferences).

Recent and historical diets in the GYE have the highest levels of energy and protein due to the availability of meat in that ecosystem. High dietary protein levels in GYE bears are consistent with their larger body size when compared with other interior North American brown bears and with their rapid rate of population recovery during the last three decades [28, 29]. Comparisons between historical [30] and recent [33] diets do reveal, however, a change in dietary protein and energy due to the loss of key foods, which may affect bear fitness and population density. During spring, the recent male diet showed a lower digestible energy and protein content than the female diet and the historical diet, which was driven by the decrease in ungulate consumption [33]. During summer, the absence of trout and decreased consumption of pine nuts has reduced the dietary digestible energy and protein content for both females and males. Trout was the main source of energy from May to mid–August in the historical diet, while the contribution of pine nuts was important from mid-August to September. Digestible energy in the recent GYE diets was dominated by terrestrial meat and green vegetation during summer. Despite the difference in the nutritional parameters between the recent and the historical diets in the GYE, Schwartz, Fortin [25] did not find clear evidence of a decline in body condition of females or population productivity during 2000–2010 [25].

However, ecosystem alterations due to human intervention might have strong consequences in bear foraging strategies and food habits. Abundant ungulates in the historical GYE was the consequence of an increased elk population following the extirpation of grey wolves (*Canis lupus*) in the early 20^th^ century [81]. Later, the re-introduction of wolves in 1995 and 1996, which now compete with brown bears for ungulate carcases, together with the reduction of trout is affecting the foraging strategies of brown bears (e.g., recently switching to consuming elk calves in the spring rather than carcass scavenging [39, 44]). More recently, the decreasing elk population, due in part to an expanding wolf population, has allowed berry-producing shrubs to proliferate [39]. Increased berry consumption in the late summer and fall by Yellowstone grizzly bears may help mitigate the loss of whitebark pine nuts. Further monitoring is required to see if, and how, dietary changes in the GYE impact bears in the future.

Bears can consume a wide variety of foods, which facilitates dietary switches when previously abundant foods disappear (e.g., ungulates or pine nuts). However, our results suggest that the loss of high quality foods may have a disproportionate effect on bear productivity when increased intake of alternative foods cannot fully replace the loss of energy or nutrients. Additionally, such foods may not be directly substitutable, because they often are composed of different macronutrients and they differ in digestibility and energy content.

There are two other environmental factors influencing the individual capacity to acquire energy and protein that create differences in population productivity. First, there are differences in the length of the growing season among ecosystems. For example, in the Flathead ecosystem nutritious bear foods were available for seven months (beginning of April to the end of October; [49]) while in the Alberta ecosystem and GYE useful foods were available for only six months [33, 50]. Also severe winter conditions in the Mountain ecosystems might delay food availability in spring while early winter conditions may further reduce food availability in the fall. Longer growing seasons benefit bears by increasing the time they can accumulate energy and protein reserves and by reducing the length of hibernation and therefore the energy and protein costs [16]. Second, environmental conditions influence food abundance in the ecosystems [7, 10]. Food abundance will limit nutrient intake depending on the functional response (i.e., foraging efficiency) and the nutritional quality of the food [5]. A genetic component might be also influencing the differences in life history traits and population densities in these ecosystems, as has been observed in other areas [82–86].

## Conclusions

Previous studies have illustrated the differences in brown bear diets and their correlation with life history traits and population densities [1, 7, 11, 18, 73]**.** However, the specific nutritional differences of brown bear diets between populations have not been previously quantified or assessed for the entire active period. This study is one of the first to compare ecosystem-specific brown bear diets based on the relative sources of digestible energy and protein for several interior populations. Noticeable differences in the nutritional parameters of brown bear diets were observed among several interior ecosystems, and the patterns observed suggest that individual body size and population density may be influenced by the availability of protein early in the season (by supporting lean mass gain and lactation) and the availability of energy late in the season (by supporting fat mass gain before hibernation).

Small changes in the availability of highly nutritious foods have important effects on the nutritional quality of bear diets, such as the reduction or loss of terrestrial meats, trout and pine nuts in the GYE. Changes in nutritional quality will have an even greater impact when food availability and foraging efficiency do not permit increased consumption of less nutritious foods to offset the reduction in nutritional quality. Due to the importance of the nutritional conditions on bear fitness [12, 73] and population productivity [39, 44], monitoring food availability and foraging and dietary patterns of brown bears should be a permanent part of management programs.

## Supporting Information

S1 File Numerical example. A numerical example of the model calculation is presented in this section. The example follows the steps and equations presented in the main manuscript.
(DOCX)
