# Supplementary material for: Assessing Nutritional Parameters of Brown Bear Diets among Ecosystems Gives Insight into Differences among Populations
Source: PLoS One. 2015 Jun 17;10(6):e0128088. doi: 10.1371/journal.pone.0128088 (PMC4470632; doi:10.1371/journal.pone.0128088)
Supplement: S1 File — A numerical example of the model calculation is presented in this section. The example follows the steps and equations presented in the main manuscript. (DOCX) [file pone.0128088.s001.docx]

**Supporting Information S1 Numerical Example**

**Article: “Assessing nutritional parameters of brown bear diets among ecosystems gives insight into differences among populations”**

Claudia López-Alfaro**^a,b^**, Sean C. P. Coogan^a,e^, Charles T. Robbins**^c^, Jennifer K. Fortin^d^,** Scott E. Nielsen**^a^**

^a^ Department of Renewable Resources, University of Alberta, 751 GSB, Edmonton, T6G 2H1, AB, Canada. Phone: 1 780 492 1656; Fax: 1 780 492 4323. e-mail: lopez@ualberta.ca; scoogan@ualberta.ca; scottn@ualberta.ca ^.^

^b^ Departamento de Ciencias Ambientales y Recursos Naturales Renovables, Universidad de Chile, Av. Santa Rosa 11315, Casilla 9206, Santiago Chile.

^c^ School of the Environment and School of Biological Sciences, Washington State University, Pullman, WA, USA. Phone: 1 509 335 1119; Fax: 1 509 335 3184. e-mail: ctrobbins@wsu.edu

**^d^ School of Biological Sciences, Washington State University, Pullman, WA, USA. Present address: US Geological Survey, Alaska Science Center, Anchorage, AK, USA. Phone: 1 907 786 7058. e-mail: jenn_fortin@hotmail.com.**

^e^ School of Biological Sciences and the Charles Perkins Centre, University of Sydney, Sydney, NSW 2006, Australia. e-mail: sean.coogan@sydney.edu.au

**SUPPORTING INFORMATION S1 NUMERICAL EXAMPLE**

A numerical example of the model calculation is presented in this section. The example follows the steps and equations presented in the main manuscript.

*Example:*

Calculations start with the results of the fecal analysis (%.FV(fi); Table S1). Fecal analysis contains three food items (fi): Green vegetation, Terrestrial meat and Fruit. For example purpose, we used a fix CFs presented in Table 1. For the nutritional values per foods categories we used the average values presented in Table 2. In the model, nutritional values are estimated randomly from a normal distribution curve. These curves are built with the average and standard deviation presented in Table 2. Due to this variation we run the model 1000 times to obtain the average values and SD. For example purpose, we used the average nutritional value presented in Table 3.

Acronyms

- FV = Fecal volume (%)
- DM = Dry matter (% of fresh matter)
- DMDig = Digestible dry matter (%)
- GrossE = Gross energy (kcal/g)
- EDig = Energy digestibility (%)
- PC = Protein content (%)
- PDig = Protein digestibility (%)

DMDig, GrossE, EDig, PC and PDig in a dry matter basis.

*First section: from Fecal Volume (%) to Digestible Dry Matter (DMDig):*

Model input:

- FV (green vegetation) = 60%
- FV (terrestrial meat) = 10%
- FV (fruit) = 30%

**Eq. 1**

- g.DMDig(green vegetation) = 60 × 0.26 ÷ 91.6 × 100 = 17.0
- g.DMDig(terrestrial meat) = 10 × 4 ÷ 91.6 × 100 = 43.7
- g.DMDig(fruit) = 30 × 1.2 ÷ 91.6 × 100 = 39.3

| ***Numerical example first section*** | | | | |
| --- | --- | --- | --- | --- |
| Food item | %. FV(fi) | *CF (fi)* | *%. FV*CF(fi)* | *g.DMDig(fi)* |
| Green vegetation | 60 | 0.26 | 15.6 | 17.0 |
| Terrestrial meat | 10 | 4.0 | 40 | 43.7 |
| Fruit | 30 | 1.2 | 36 | 39.3 |
| Sum = | 100 |  | 91.6 | 100 |
| CF(fi) can be fix or varied randomly among its range presented in Table 1. | | | | |

***Second section: from Digestible Dry Matter intake (DMDig) to Fresh Food Intake:***

**Eq.2** $g.FFood(f)=g.DMDig(f)\div\left(\%.DM(f)\times\%.DMDig(f)\right)\times10000$

- g.FFood(green vegetation) = 17.0 ÷ (20.1 × 36.6) × 10000 = 231.5
- g.FFood(terrestrial meat) = 43.7 ÷ (27.4 × 87.5) × 10000 = 182.1
- g.FFood(fruit) = 39.3 ÷ (15.1 × 63.9) × 10000 = 407.3

**Eq.3**

- g.FFDiet(green vegetation) = 231.5 ÷ 821.0 × 1000 = 280.0
- g.FFDiet(green vegetation) = 182.1 ÷ 821.0 × 1000 = 221.9
- g.FFDiet(green vegetation) = 407.3 ÷ 821.0 × 1000 = 496.1

| *Numerical example second section* | | | | | |
| --- | --- | --- | --- | --- | --- |
| Food item | *g.DMDig(fi)* | *%.DM(fi)* | *%.DMDig(fi)* | *g.Ffood(fi)* | *g.FFDiet(fi)* |
| Green vegetation | 17.0 | 20.1 | 36.6 | 231.5 | 282.0 |
| Terrestrial meat | 43.7 | 27.4 | 87.5 | 182.1 | 221.9 |
| Fruit | 39.3 | 15.1 | 63.9 | 407.3 | 496.1 |
| Sum = | 100 |  |  | 821.0 | 1000.0 |
| %.DM(fi) and DMDig(fi) varied randomly from a normal distribution curve. These curves are built with the average and standard deviation presented in Table 2. | | | | | |

***Third section: estimations of digestible energy and digestible protein in one kilogram of fresh diet.***

**Eq.4** $kcal.DigestibleE(f)=g.FFDiet(f)\times\%.DM(f)\times kcal/g.GrossE(f)\times\%.EDig(f)\div10000$

- Kcal.DigestibleE(green vegetation) = 282.0 × 20.1 × 4.5 × 41.3 ÷ 10000 = 105.3
- Kcal.DigestibleE(terrestrial meat) = 221.9 × 27.4 × 5.2 × 92.5 ÷ 10000 = 292.4
- Kcal.DigestibleE(fruit) = 496.1 × 15.1 × 4.3 × 60.0 ÷ 10000 = 193.3

**Eq.5**

- Kcal. DigestableE(diet) = 105.3 + 292.4 + 193.3 = 591.0

| *Numerical example third section. Estimation of digestible energy* | | | | | |
| --- | --- | --- | --- | --- | --- |
| Food item | g.FFDiet(f) | %.DM(f) | Kcal.GrossE(f) | %.EDig(f) | Kcal.DigestibleE(f) |
| Green vegetation | 282.0 | 20.1 | 4.5 | 41.3 | 105.3 |
| Terrestrial meat | 221.9 | 27.4 | 5.2 | 92.5 | 292.4 |
| Fruit | 496.1 | 15.1 | 4.3 | 60.0 | 193.3 |
| Sum = | 1000.0 |  |  |  | 591.0 |
| %.DM(f); kcal.GrossE(f); %.EDig(f) varied randomly from a normal distribution curve. These curves are built with the average and standard deviation presented in Table 2. | | | | | |

**Eq. 6** $g.DigestibleP(f)=g.FFDiet(f)\times\%.DM(f)\times\%.PC(f)\times\%.PDig(f)\div1000000$

- g.DigestibleP(green vegetation) = 282.2 × 20.1 × 25.9 × 74.5 ÷ 1000000 = 10.9
- g.DigestibleP(terrestrial meat) = 221.9 × 27.4 × 72.9 × 88.2 ÷ 1000000 = 39.1
- g.DigestibleP(fruit) = 496.1 × 15.1 × 4.6 × 14.8 ÷ 1000000 = 0.5

**Eq.7**

- g. DigestableP(diet) = 10.9 + 39.1 + 0.5 = 50.5

| *Numerical example third section. Estimation of digestible protein* | | | | | |
| --- | --- | --- | --- | --- | --- |
| Food item | g.FFDiet(f) | %.DM(f) | %.PC(f) | %.PDig(f) | g.DigestibleP(f) |
| Green vegetation | 282.0 | 20.1 | 25.9 | 74.5 | 10.9 |
| Terrestrial meat | 221.9 | 27.4 | 72.9 | 88.2 | 39.1 |
| Fruit | 496.1 | 15.1 | 4.6 | 14.8 | 0.5 |
| Sum = | 1000.0 |  |  |  | 50.5 |
| %.DM(f); %.PC(f); %.PDig(f) varied randomly from a normal distribution curve. These curves are built with the average and standard deviation presented in Table 2. | | | | | |
